# Supplementary material for: Human VMPFC encodes early signatures of confidence in perceptual decisions
Source: eLife. 2018 Sep 24;7:e38293. doi: 10.7554/eLife.38293 (PMC6199131; doi:10.7554/eLife.38293)
Supplement: Supplementary file 1. [file elife-38293-supp1.docx]

**Supplementary Tables**

|  |  |  | Peak MNI coordinates (mm) | | |  |
| --- | --- | --- | --- | --- | --- | --- |
| **Brain region** | **BA** | **Laterality** | **X** | **Y** | **Z** | **Z value (peak)** |
| **Positive parametric effect (Z > 2.57)** |  |  |  |  |  |  |
| Striatum (nucleus accumbens / ventral putamen) | - | L | -10 | 4 | -10 | 4.64 |
|  | - | R | 12 | 4 | -10 | 4.09 |
| Lateral orbitofrontal cortex | 11/47 | L | -28 | 46 | -8 | 4.46 |
|  | 47 | R | 32 | 38 | -6 | 3.86 |
| Anterior cingulate cortex | 32/10 | R, L | 2 | 36 | 6 | 4.19 |
| Lateral occipital cortex (inferior) | 19 | L | -42 | -68 | -10 | 4.04 |
|  | 19 | R | 48 | -82 | 8 | 3.13 |
| Middle frontal gyrus (anterior) | 10 | R | 40 | 62 | 10 | 3.94 |
| Striatum (dorsal putamen / pallidum) | - | L | -28 | -18 | 2 | 3.72 |
| Occipital pole | 17 | R, L | 4 | -102 | 8 | 3.66 |
| Cerebellum | - | R | 22 | -46 | -22 | 3.55 |
| Inferior temporal gyrus | 37 | R | 54 | -46 | -16 | 3.51 |
| **Negative parametric effect (Z < -2.57)** |  |  |  |  |  |  |
| Superior frontal gyrus (supplementary motor area) | 6 | R | 14 | 12 | 64 | 5.62 |
| Dorsomedial prefrontal cortex | 6/32 | R, L | -6 | 12 | 52 | 4.13 |
| Inferior frontal gyrus | 44/45 | R | 50 | 16 | 2 | 3.95 |
| Precentral gyrus | 6 | R | 50 | 4 | 46 | 3.61 |
|  | 6 | L | -44 | 2 | 38 | 3.46 |
| MNI, Montreal Neurological Institute; L, left hemisphere; R, right hemisphere; BA, approximate Broadmann area. | | | | | | |

**Supplementary file 1A**. Complete list of brain activations correlating with subjects’ confidence reports, at the time of stimulus onset (decision phase).

|  |  |  | Peak MNI coordinates (mm) | | |  |
| --- | --- | --- | --- | --- | --- | --- |
| **Brain region** | **BA** | **Laterality** | **X** | **Y** | **Z** | **Z value (peak)** |
| **Positive parametric effect (Z > 2.57)** |  |  |  |  |  |  |
| Amygdala / Hippocampus | - | R | 28 | -10 | -14 | 4.16 |
|  | - | L | -28 | -12 | -12 | 3.27 |
| Putamen | - | L | -22 | 18 | 2 | 4.01 |
| Precentral gyrus | 6/4 | L | -38 | -10 | 70 | 3.87 |
|  | 6 | R | 38 | -14 | 70 | 3.04 |
| **Negative parametric effect (Z < -2.57)** |  |  |  |  |  |  |
| Angular gyrus | 39 | L | -58 | -56 | 34 | 5.87 |
| Angular gyrus | 39 | R | 60 | -54 | 36 | 5.82 |
| Superior frontal gyrus / RLPFC | 10/9 | R | 24 | 58 | 26 | 5.84 |
|  | 10/9 | L | -20 | 52 | 26 | 5.2 |
| Inferior frontal gyrus (orbital area) / Anterior insula | 13/45 | L | -44 | 24 | -8 | 5.58 |
|  | 13/45 | R | 42 | 22 | -6 | 5.26 |
| Middle frontal gyrus | 8/9 | R | 44 | 20 | 42 | 5.56 |
|  | 8/9 | L | -40 | 20 | 42 | 4.92 |
| Medial frontal gyrus | 8/9 | L, R | 0 | 42 | 34 | 5.19 |
| Inferior frontal gyrus (triangular area) | 45 | L | -50 | 22 | 6 | 5.02 |
|  | 45 | R | 58 | 30 | 8 | 4.94 |
| Precuneus | 7 | L, R | -2 | -68 | 38 | 4.51 |
| Occipitotemporal gyrus | 37 | L | -38 | -62 | -22 | 4.34 |
| Posterior cingulate cortex | 23 | L, R | -2 | -26 | 32 | 4.76 |
| Middle temporal gyrus (anterior) | 20/21 | R | 50 | 2 | -34 | 4.36 |
| Thalamus | - | R | 10 | -10 | 2 | 4.35 |
|  | - | L | -12 | -10 | 6 | 3.82 |
| Lingual gyrus | 18 | L | -2 | -80 | 0 | 4.14 |
| Calcarine cortex | 17 | R | 16 | -90 | 2 | 4.14 |
|  | 17 | L | -12 | -92 | 2 | 3.93 |
| Middle temporal gyrus (posterior) | 21/37 | R | 56 | -34 | -12 | 3.93 |
|  | 21 | L | -54 | -30 | -8 | 3.82 |
| Inferior occipital gyrus | 18 | R | 28 | -90 | -10 | 3.19 |
| Lateral occipital cortex (superior) | 19 | R | 44 | -74 | 20 | 3.58 |
|  | 19 | L | -40 | -88 | 20 | 3.43 |
| MNI, Montreal Neurological Institute; L, left hemisphere; R, right hemisphere; BA, approximate Broadmann area; RLPFC, rostrolateral prefrontal cortex | | | | | | |

**Supplementary file 1B.** Complete list of brain activations correlating with subjects’ confidence reports, at the time of confidence rating (rating phase).
