# Supplementary material for: Human VMPFC encodes early signatures of confidence in perceptual decisions
Source: eLife. 2018 Sep 24;7:e38293. doi: 10.7554/eLife.38293 (PMC6199131; doi:10.7554/eLife.38293)
Supplement: Transparent reporting form [file elife-38293-transrepform.docx]

***eLife’s* transparent reporting form**

We encourage authors to provide detailed information *within their submission* to facilitate the interpretation and replication of experiments. Authors can upload supporting documentation to indicate the use of appropriate reporting guidelines for health-related research (see [EQUATOR Network](http://www.equator-network.org/%20)), life science research (see the [BioSharing Information Resource](https://biosharing.org/)), or the [ARRIVE guidelines](http://www.plosbiology.org/article/info:doi/10.1371/journal.pbio.1000412) for reporting work involving animal research. Where applicable, authors should refer to any relevant reporting standards documents in this form.

If you have any questions, please consult our Journal Policies and/or contact us: [editorial@elifesciences.org](mailto:editorial@elifesciences.org).

**Sample-size estimation**

- You should state whether an appropriate sample size was computed when the study was being designed
- You should state the statistical method of sample size computation and any required assumptions
- If no explicit power analysis was used, you should describe how you decided what sample (replicate) size (number) to use

Please outline where this information can be found within the submission (e.g., sections or figure legends), or explain why this information doesn’t apply to your submission:

No explicit power analysis was conducted for determining sample size. This was estimated conservatively (N=30) on the basis of effect sizes in simultaneous EEG/fMRI datasets acquired in our lab (Fouragnan et al., 2015; Pisauro et al., 2017). Notably, our EEG analysis was performed on individual subjects using cross validation, such that in estimating our electrophysiologically-derived measure of confidence, each subject became their own replication unit (Smith and Little, 2018).

Relevant information is included in *Materials and methods* (*Participants* section, p. 26).

**Replicates**

- You should report how often each experiment was performed
- You should include a definition of biological versus technical replication
- The data obtained should be provided and sufficient information should be provided to indicate the number of independent biological and/or technical replicates
- If you encountered any outliers, you should describe how these were handled
- Criteria for exclusion/inclusion of data should be clearly stated
- High-throughput sequence data should be uploaded before submission, with a private link for reviewers provided (these are available from both GEO and ArrayExpress)

Please outline where this information can be found within the submission (e.g., sections or figure legends), or explain why this information doesn’t apply to your submission:

No replications were performed in this study.

Information about removal of outlier subjects can be found in *Materials and methods* (*Participants* section, p. 26). No outlier removal was applied at the within-subject (i.e., trial) level of analysis.

**Statistical reporting**

- Statistical analysis methods should be described and justified
- Raw data should be presented in figures whenever informative to do so (typically when N per group is less than 10)
- For each experiment, you should identify the statistical tests used, exact values of N, definitions of center, methods of multiple test correction, and dispersion and precision measures (e.g., mean, median, SD, SEM, confidence intervals; and, for the major substantive results, a measure of effect size (e.g., Pearson's r, Cohen's d)
- Report exact p-values wherever possible alongside the summary statistics and 95% confidence intervals. These should be reported for all key questions and not only when the p-value is less than 0.05.

Please outline where this information can be found within the submission (e.g., sections or figure legends), or explain why this information doesn’t apply to your submission:

Subject-averaged Pearson’s R statistics are reported for within-subject correlation analyses. The correlation coefficient reported in *Results*, *Dynamic model of decision making* section (p. 14, depicted in Fig. 3D) was obtained using the more robust, percentage bend correlation analysis (Wilcox, 1994). Group-level significance of correlation coefficients was assessed using one-sample t-tests. Relevant statistical tests are reported in *Results* (*Behaviour* and *EEG-derived measure of confidence* sections, p. 5/6 and p. 10/12, respectively).

Subject-averaged R^2^ statistics are reported for within-subject serial autocorrelation regression analyses (see *Results, Behaviour* and *EEG-derived measure of confidence* sections, p. 7 and 11, respectively)

Quantification and significance thresholding of the EEG multivariate classification results are described in *Materials and methods* (*Single-trial EEG analysis* section, p. 32/33).

Information about statistical analysis and correction for multiple comparisons of the fMRI data is found in *Materials and methods* (*Resampling procedure for fMRI thresholding* section, p. 40).

Exact p-values are reported for all statistical tests in *Results* (p. 5-20), except for cases where these are trivially small, in which case we report them as p<0.001.

Single-subject data is shown for behavioural (Fig. 1, p. 6), EEG (Fig. 2, p. 8), and computational modeling results (Fig. 3, p. 14, see associated supplementary figures).

(For large datasets, or papers with a very large number of statistical tests, you may upload a single table file with tests, Ns, etc., with reference to sections in the manuscript.)

**Group allocation**

- Indicate how samples were allocated into experimental groups (in the case of clinical studies, please specify allocation to treatment method); if randomization was used, please also state if restricted randomization was applied
- Indicate if masking was used during group allocation, data collection and/or data analysis

Please outline where this information can be found within the submission (e.g., sections or figure legends), or explain why this information doesn’t apply to your submission:

N/A

**Additional data files (“source data”)**

- We encourage you to upload relevant additional data files, such as numerical data that are represented as a graph in a figure, or as a summary table
- Where provided, these should be in the most useful format, and they can be uploaded as “Source data” files linked to a main figure or table
- Include model definition files including the full list of parameters used
- Include code used for data analysis (e.g., R, MatLab)
- Avoid stating that data files are “available upon request”

Please indicate the figures or tables for which source data files have been provided:

Source data files are provided for all figures and tables, as Matlab code and data.
